# Modulation of the Thrombin Pathway Restores LTP in a Pilocarpine Mice Model of Status Epilepticus

**DOI:** 10.3389/fncel.2022.900925

**Published:** 2022-05-24

**Authors:** Efrat Shavit-Stein, Shani Berkowitz, Tal Davidy, Uri Fennig, Shani Guly Gofrit, Amir Dori, Nicola Maggio

**Affiliations:** ^1^Department of Neurology, The Chaim Sheba Medical Center, Ramat Gan, Israel; ^2^Department of Neurology and Neurosurgery, Sackler Faculty of Medicine, Tel Aviv University, Tel Aviv, Israel; ^3^The TELEM Rubin Excellence in Biomedical Research Program, The Chaim Sheba Medical Center, Ramat Gan, Israel; ^4^Talpiot Medical Leadership Program, The Chaim Sheba Medical Center, Ramat Gan, Israel; ^5^Sagol School of Neuroscience, Tel Aviv University, Tel Aviv, Israel

**Keywords:** thrombin, PAR1, hippocampus, pilocarpine, seizures, status epilepticus

## Abstract

**Background:**

Status epilepticus (SE) leads to memory impairment following a seizure, attributed to long-term potentiation (LTP) reduction. Thrombin, a coagulation factor that activates protease-activated receptor 1 (PAR1) is involved in cognitive impairment following traumatic brain injury by reducing hippocampal LTP and in seizures as seen in a SE pilocarpine-induced mice model. Thrombin pathway inhibition prevents this cognitive impairment. We evaluated the effect of thrombin pathway inhibition in the pilocarpine-induced SE mice model, on LTP, hippocampal, and serum markers for inflammation, the PAR1 pathway, and neuronal cell damage.

**Methods:**

SE was induced by injecting C57BL/6J mice with pilocarpine. Before pilocarpine injection, mice were injected with either the specific thrombin inhibitor α-NAPAP [Nα-(2-naphthalene-sulfonylglycyl)-4-amidino-DL-phenylalaninepiperidide], the PAR1 antagonist SCH79797, or vehicle-only solution. Recordings of excitatory postsynaptic potentials (EPSP) were conducted from hippocampal slices 24 h following pilocarpine injection. Hippocampal real-time PCR for the quantification of the PAR1, prothrombin, and tumor necrosis factor α (TNF-α) mRNA expression levels was conducted. Serum levels of neurofilament light chain (NfL) and TNF-α were measured by a single molecule array assay.

**Results:**

The EPSP was reduced in the pilocarpine-induced SE mice (*p* < 0.001). This reduction was prevented by both NAPAP and SCH79797 treatments (*p* < 0.001 for both treatments). Hippocampal expression of TNF-α was elevated in the pilocarpine-induced SE group compared to the control (*p* < 0.01), however, serum levels of TNF-α were not changed. NfL levels were elevated in the pilocarpine-induced SE group (*p* = 0.04) but not in the treated groups.

**Conclusions:**

Pilocarpine-induced SE reduces LTP, in a thrombin PAR1-related mechanism. Elevation of serum NfL supports neuronal damage accompanying this functional abnormality which may be prevented by PAR1 pathway modulation.

## Introduction

Convulsive Status epilepticus (SE) affects approximately 24/100,000 patients per year (Leitinger et al., 2019). SE causes high mortality rates, with an extensive functional impairment in the following days and months (Legriel et al., 2010). Retrograde amnesia following a seizure is a well-known phenomenon of the convulsing brain, as can be seen following electroconvulsive therapy (Squire et al., 1976). In animals, SE can be modeled using direct cholinergic agonists such as pilocarpine, which induces seizures by both systemic and intracerebral administration (Turski et al., 1989). Impaired learning and memory following seizures are evident in animal models for SE as well, as demonstrated by reduced novel object recognition and performance in the water maze in the pilocarpine and kainic acid models (Pearson et al., 2014). In an undeveloped brain, pilocarpine-induced SE leads to memory impairment noted 3 months following the insult and an increased epileptogenic tendency (Kubová et al., 2004).

The mechanism by which SE impairs learning is thought to involve a reduction in long-term potentiation (LTP) in an N-methyl-D-aspartate receptor (NMDAR) dependent- mechanism (Kryukov et al., 2016). Changes in NMDAR subunits in hippocampal neurons following pentylenetetrazole-induced SE, a GABA receptor antagonist, further support NMDAR involvement (Postnikova et al., 2017). In the pilocarpine SE-induced model, the underlying mechanism also involves the modification of AMPA receptors (Cruz Del Angel et al., 2021), weakening of the NMDAR-dependent signaling (Postnikova et al., 2021), and the reduction of synaptopodin (an actin-binding protein), which regulates the ability of neurons to express synaptic plasticity (Lenz et al., 2017).

The coagulation factor thrombin and its protease-activated receptor 1 (PAR1) are pivotal in neuronal pathologies, including inflammation and degeneration (Gofrit and Shavit-Stein, 2019; Festoff and Citron, 2019). Penetration of serum thrombin into the CNS following traumatic brain injury is well known to induce seizures (Lee et al., 1997). Furthermore, thrombin is involved in cognitive dysfunction following minimal traumatic brain injury (mTBI) in animal models, *via* enhancement of hippocampal neuronal reactivity, which causes an impaired ability to undergo LTP. The thrombin excitatory effect is PAR1 mediated and involves NMDAR activation (Maggio et al., 2008; Isaeva et al., 2012). Blocking PAR1 restores the LTP and protects against mTBI-induced amnesia (Itzekson et al., 2014).

During SE, the permeability of the blood-brain barrier (BBB) changes, allowing penetration of serum proteins (Gorter et al., 2015), including thrombin. Experimental models for SE show elevation of brain thrombin activity following SE, accompanied by elevation of electrical activity in hippocampal CA1 and CA3 neurons, an effect that is blocked by a PAR1 antagonist (Golderman et al., 2019). Recent work by our laboratory shows elevated hippocampal thrombin activity in pilocarpine-induced SE, with behavioral improvement in animals treated with systemic thrombin inhibition (Lenz et al., 2019). These results led us to study the effect of systemic thrombin inhibition on LTP in a pilocarpine-induced SE animal model. Following pilocarpine-induced SE, excitatory postsynaptic potentials (EPSP) were reduced. EPSP was normalized by prior systemic thrombin inhibition with the direct thrombin inhibitor NAPAP, as well as with selective blocking of PAR1 by the antagonist SCH79797. Following pilocarpine-induced SE, hippocampal tumor necrosis factor α (TNF-α) expression was elevated, as well as serum levels of neurofilament light chain (NfL), supporting local brain inflammation together with neuronal damage. The increase of both the local inflammatory and the systemic neuronal damage markers was prevented by the PAR1 pathway modulations.

## Methods

### Animals and Seizure Rating

Animal handling was approved by the Institutional Animal Care and Use Committee at the Chaim Sheba Medical Center (#1089-17, #736-12), which adheres to the national law. Every effort was made to minimize the distress and pain of animals. Briefly, SE was induced in 3-month-old male C57BL/6J mice by a single intraperitoneal (i.p.) injection of 250 mg/kg pilocarpine (Sigma, P6503-5G) hydrochloride as previously described (Lenz et al., 2017). To avoid side effects induced by peripheral cholinergic activation, mice were treated with atropine methyl nitrate (1 mg/kg, i.p. Sigma SML0732) 30 min before pilocarpine injection, while diazepam (3 mg/kg, i.p. Assival Teva 10 mg/2 ml 289716.02-IL) was used to halt convulsions. All animals received diazepam (3 mg/kg, i.p.) 90 min after pilocarpine injection. After the pilocarpine injection, behavioral seizure activity was documented every 10 min by an investigator blind to the experimental conditions using a modified Racine’s scale. Seizure activity was scored as follows: 0-no seizure activity, 1-freezing, 2-single twitches, 3-orofacial seizures, 4-clonic seizures, 5-tonic seizures, 6-death (Lenz et al., 2019). Behavioral assessment was immediately discontinued if animals reached stage 5, i.e., tonic seizures persisted and then diazepam was injected (3 mg/kg; i.p.) to halt seizures. All groups including the control group received the same treatment with diazepam. The vehicle-only group received vehicle + pilocarpine.

### Systemic Thrombin and PAR1 Inhibition

All pharmacological substances were dissolved according to manufacturers’ instructions. To test the effects of thrombin or PAR1 inhibition, animals were i.p. injected with a single dose of the thrombin inhibitor α-NAPAP [Nα-(2-naphthalene-sulfonyl glycyl)-4-amidino-DL-phenylalaninepiperidide, Santa Cruz, 0.75 mg/kg in phosphate-buffered saline (PBS), SC-208083] or the PAR1 antagonist SCH79797 (Tocris Bioscience, 1592, 25 μg/kg in PBS, prepared immediately before use) 15 min before the atropine injection. All animals received the same volume of vehicle-only solutions as a control for this intervention.

### Electrophysiology

Animals were used for the recording experiments 24 h after pilocarpine injections. They were rapidly decapitated and 350 μm coronal dorsal hippocampal slices were used. Slices were incubated for 1.5 h in a humidified, carbonated (5% CO_2_ and 95% O_2_) gas atmosphere at 33 ± 1°C and were perfused with artificial CSF [containing (in mM) 124 NaCl, 2 KCl, 26 NaHCO_3_, 1.24 KH_2_PO_4_, 2.5 CaCl_2_, 2 MgSO_4_ and 10 glucose, pH 7.4] in a standard interface chamber. To avoid hyperexcitability, a cut between CA3 and CA1 was made before the experiments. Recordings were made with a glass pipette containing 0.75 M NaCl (4 MΩ) placed in the stratum radiatum CA1. Stimulation was evoked using a Master 8 pulse stimulator (A.M.P.I., Jerusalem, Israel) and was delivered through a bipolar nichrome electrode. LTP was induced by high-frequency stimulation consisting of 100 pulses at twice the test intensity, delivered at a frequency of 100 Hz (HFS; 100 Hz, 1 s). Before and after the tetanic stimulation, stimulation was applied at a frequency of 0.033 Hz. Responses were digitized at 5 kHz and stored on a computer. Off-line analysis and data acquisition were performed using Spike 2 software (Spike2 Software, RRID: SCR_000903 CED, Cambridge, England). All numerical data are expressed as mean ± SEM, and EPSP slope changes after tetanic stimulation were calculated with respect to baseline. There were no systematic differences in the magnitudes of the baseline responses in the different conditions. All values reported refer to 30 min after tetanic stimulation. Each experimental group consisted of eight mice in total, and a hippocampal slice from each animal was taken for measurement.

### Coagulation and Inflammation Protein Expression in the Hippocampus

Prior to the harvest, the animals were anesthetized with pentobarbital [CTS, (0.8 mg/kg)]. The brains were removed, and the hippocampi were dissected. Hippocampal mRNA was extracted by lysis buffer addition accordingly to the Bio-Rad Aurum 732-6820 kit (Bio-Rad Laboratories, Hercules, CA, USA). One microgram of total RNA was used for reverse transcription using a high-capacity cDNA reverse transcription kit (Applied Biosystems). The quantitative real-time polymerase chain reaction was performed on the StepOne^TM^ Real-Time PCR System (Applied Biosystems, Rhenium, Israel, RRID: SCR_014281) using Fast SYBR Green Master (ROX; Applied Biosystems). Hypoxanthine guanine phosphoribosyltransferase served as a reference gene in this analysis (primer list). A standard amplification program was used [1 cycle of 95°C for 20 s and 40 cycles of 95°C for 3 s and 60°C for 30 s]. The primers used in this analysis are listed in Table 1. Five replicates were performed for each animal. The results were normalized to the reference gene expression within the same cDNA sample and calculated using the ΔCt method with results reported as fold changes relative to the hippocampi of control animals and reported as mean ± SE.

**Table 1:** List of primers used to assess coagulation and inflammation mRNA expression.

Gene	Forward	Reverse
PAR1	GCCTCCATCATGCTCATGAC	AAAGCAGACGATGAAGATGCA
PT	CCGAAAGGGCAACCTAGAGC	GGCCCAGAACACGTCTGTG
TNF-α	GACCCTCACACTCAGATCATCTTCT	CCTCCACTTGGTGGTTTGCT
HPRT	GATTAGCGATGATGAACCAGGTT	CCTCCCATCTCCTTCATGA CA

*PAR1, protease-activated receptor 1; PT, prothrombin; TNF-α, tumor necrosis factor α; HPRT, Hypoxanthine guanine phosphoribosyltransferase*.

### Neurofilament Light Chain and TNF-α Measurement in the Serum

Mice were anesthetized and blood samples were collected. The collected blood was incubated at room temperature for 30 min to enable clot formation and centrifuged at 1,500 *g* for 10 min at room temperature. The serum was frozen and stored at −80°C until analysis. For NfL measurement sera were initially diluted at 1:1,000. When an obtained concentration was higher than 500 pg/ml, an additional dilution of 1:5,000 was further tested. For TNF-α, sera samples were diluted 1:8. NfL and TNF-α concentrations were measured in duplicates by a single molecule array (Simoa) assay (Quanterix, Boston, MA) employing commercial kits (NF-light Advantage kit and mouse TNF-α discovery kit for HD-1/HD-X adjusted for SR-X, UmanDiagnostics Umea, Sweden), using a bead-conjugated immunocomplex. The immunocomplex was applied to a multi-well array designed to enable imaging of every single bead. The average number of enzymes per bead (AEB) of each sample was interpolated onto the calibrator curve constructed by AEB measurements on commercial NfL and TNF-α (UmanDiagnostics), serially diluted in an assay diluent. Samples were analyzed using one batch of reagents. Animal treatment information was blinded to the investigator performing both the assay and the analysis.

### Statistical Analysis

Statistical analyses and graphs were conducted using GraphPad Prism (version 8.00 for Windows, GraphPad Software, La Jolla California USA^1^). Flowing normality was evaluated by D’Agostino-Pearson. Unpaired *t*-tests, one-way ANOVA, and two-way ANOVA followed by a *post-hoc* test were applied on normally distributed data sets. One-way ANOVA was followed by either Dunnett’s or Tukey’s *post-hoc* analyses. Two-way ANOVA was followed by Sidak’s *post-hoc* analysis. Mann Whitney, or Kruskal-Wallis tests were applied on non-normal distributed data sets. Results are expressed as mean ± SEM, *p* values < 0.05 were considered significant.

## Results

### Seizure Severity and Electrophysiology

**Figure 1:**
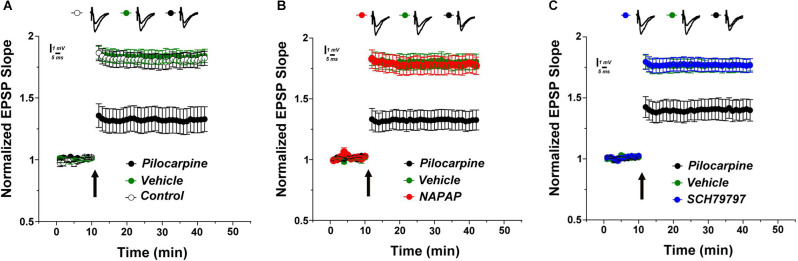
Lower LTP in pilocarpine injected animals is prevented by specific thrombin inhibition. **(A)** LTP was significantly reduced in pilocarpine treated animals compared to control. Both the specific thrombin inhibitor NAPAP **(B)** and the PAR1 inhibitor SCH79797 **(C)** prevented LTP reduction (*n* = 8 for each group).

The rating of seizure activity by the modified Racine’s scale revealed a lower score in the treated groups as indicated by the significant interaction between the time and the treatment groups (*F*_(16, 168)_ = 3.346, *p* < 0.0001). Dunnett’s *post-hoc* analysis showed that both NAPAP and SCH 79797 significantly improve the seizure score compared to control, beginning at 50 min following the seizure (2.12 ± 0.1, 1.5 ± 0.2, 3.5 ± 0.3, respectively, *p* < 0.01 for both treatments). The LTP measured 24 h after a pilocarpine injection resulted in a lower LTP compared to the one evoked in controls (untreated animals) or vehicle-treated animals. At 30 min following tetanic stimulations, EPSP values in the pilocarpine-induced SE mice were 1.32 ± 0.105 compared to 1.83 ± 0.051 (controls) and to 1.81 ± 0.056 (vehicle-treated animals, Figure 1A; *F*_(2, 21)_ = 14.9; *p* < 0.001). Strikingly, the thrombin inhibitor, α-NAPAP injected 45 min before pilocarpine prevented the decline of the evoked LTP caused by pilocarpine. Indeed, at 30 min following tetanic stimulations EPSP values were 1.32 ± 0.083, 1.80 ± 0.065 and 1.78 ± 0.075, respectively for pilocarpine, α-NAPAP and vehicle groups (Figure 1B; *F*_(2, 21)_ = 13.1, *p* < 0.001). Similarly, the PAR1- antagonist injected prior to pilocarpine blocked the effect of pilocarpine on LTP. Specifically, at 30 min following tetanic stimulations EPSP values were 1.40 ± 0.082, 1.76 ± 0.054 and 1.77 ± 0.052, respectively for pilocarpine, PAR1-antagonist and vehicle groups (Figure 1C; *F*_(2, 21)_ = 10.3, *p* < 0.001). Altogether these data show that pilocarpine blocks LTP likely through a thrombin, PAR1 mediated mechanism.

### Hippocampal Gene Expression of Coagulation and Inflammatory Factors

**Figure 2:**
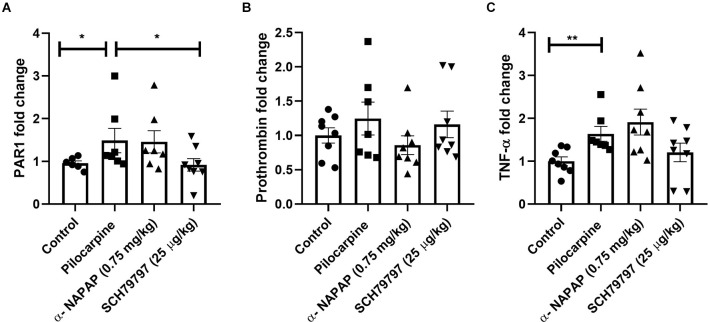
Coagulation and inflammation mRNA expression modification in the hippocampus of animals 24 h after SE induction. **(A)** PAR1 levels increased significantly in the pilocarpine-only group compared to the SCH79797 PAR1 antagonist group. No significant differences between groups were measured in prothrombin **(B)** relative expression. Levels of the inflammatory marker TNF-α **(C)** were elevated following SE induction in the pilocarpine-only group compared to controls (Coagulation and inflammatory markers: control *n* = 8, pilocarpine *n* = 7, α-NAPAP *n* = 8, SCH79797 *n* = 7). SE, status epilepticus; PAR1, protease-activated receptor; TNF-α, tumor necrosis factor α. *p < 0.05, **p < 0.01.

Hippocampal coagulation mRNA expression was assessed. A significant increase in PAR1 mRNA was found in the pilocarpine-induced SE mice compared to controls (1.49 ± 0.28, 0.96 ± 0.05, respectively, *p* = 0.03, Mann-Whitney, Figure 2A). Interestingly, the treatment with SCH79797 significantly prevented this increase and even reduced hippocampal PAR1 expression in pilocarpine-induced SE mice (0.92 ± 0.15, 1.49 ± 0.28, respectively, *p* = 0.02, Mann-Whitney, Figure 2A). Prothrombin levels did not significantly differ between any of the groups (*p* = 0.44, Kruskal-Wallis). Levels of prothrombin in the pilocarpine only group were similar to the controls, the α-NAPAP and the PAR1 antagonist groups (1.25 ± 0.24, 1 ± 0.11, 0.86 ± 0.13 1.16 ± 0.19, respectively, Figure 2B). Hippocampal inflammatory TNF-α gene expression was different between the groups (*p* = 0.01, Kruskal-Wallis). TNF-α was significantly increased in the pilocarpine-only animals compared to controls (1.63 ± 0.17, 1 ± 0.10, respectively, *p* < 0.01, Mann-Whitney, Figure 2C). TNF-α expression was not affected by α-NAPAP (1.91 ± 0.30, *p* = 0.43, Mann-Whitney) or PAR1 antagonist (1.20 ± 0.21, *p* = 0.14, Mann-Whitney).

### Serum Markers of Neuronal Damage and Inflammation

**Figure 3:**
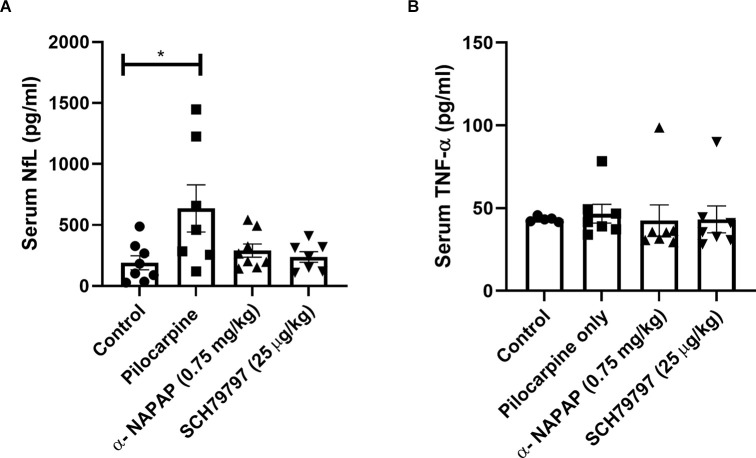
Elevation in serum marker for neuronal damage, prevented by thrombin PAR1 pathway inhibition. **(A)** Serum levels of NfL, a marker for neuronal damage, were elevated in pilocarpine only animals. Thrombin PAR1 pathway inhibitors prevented the increased NfL levels. **(B)** Systemic inflammation as evaluated by TNF-α levels was similar in all four groups. *p < 0.05.

Serum NfL levels, which are known to provide evidence for neuronal damage, were significantly elevated in the pilocarpine-induced SE mice compared to controls (635.6 ± 193.6, 190.1 ± 56.92 pg/ml, respectively, *p* = 0.04, Mann-Whitney, Figure 3A). Both NAPAP and SCH79797 prevented this NfL levels elevation which was found similar to the control (290.2 ± 54.02, 237.2 ± 43.52 pg/ml, respectively, *p* = 0.52, *p* > 0.99, respectively, Kruskal-Wallis).

TNF-α levels in the serum, representing systemic inflammation, were not significantly different between any of the four groups 24 h after pilocarpine injection (*p* = 0.13, Kruskal-Wallis, Figure 3B). Specifically, TNF-α levels in the pilocarpine-only group were similar to controls (46.59 ± 5.67, 43.26 ± 0.72 pg/ml, respectively, *p* > 0.99).

TNF-α levels in the NAPAP and PAR1 antagonist treatment groups were similar to the levels measured in the pilocarpine-only group (42.49 ± 9.41, 43.18 ± 8.08 pg/ml, *p* = 0.22, *p* = 0.58 respectively, Kruskal-Wallis).

## Discussion

In this work, we show that SE induced by pilocarpine may block LTP through a thrombin-PAR1 mediated mechanism. In addition, inflammatory brain TNF-α expression was significantly elevated following SE, without peripheral changes in protein levels. A striking effect was observed in the serum concentration of neurofilaments, which are novel markers of brain damage (Lambertsen et al., 2020; Yuan and Nixon, 2021) and neuronal damage (Gaetani et al., 2019; Barro and Zetterberg, 2021). Through these experiments, we have shown that in an animal model of SE, neurofilaments’ concentration increases in a relatively short time frame. While it is yet to be established whether neurofilament concentration rises in humans upon SE, our data may support a possible role of neurofilaments concentration as a biomarker of functional and morphological neuronal damage upon SE. The correlation between the increase in neurofilaments concentration, morphological changes, and functional impairment warrants further study.

It is interesting to note that although TNF-α mRNA expression was elevated in the hippocampus following SE, no changes in peripheral inflammatory markers, i.e., serum TNF-α protein concentration was detected. In previous studies, concentrations of TNF-α were increased in the serum of patients immediately following seizure (Sinha et al., 2008). In contrast, in an animal model, following SE, TNF-α only rose days after seizure induction (Gouveia et al., 2015). The fact that in our hands, we were not able to find such changes may lie in that TNF-α concentrations were studied early after SE. Possibly, assessing TNF-α levels in a later time frame would have shown a different effect.

The evidence that pilocarpine blocks LTP is not new. Pilocarpine is involved in LTP-related mechanisms, including a reduction in the actin-binding protein synaptopodin (Lenz et al., 2017), and changes in the expression of a-amino-3-hydroxy-5-methyliisoxazple-4-propionic acid receptors (AMPAR; Cruz Del Angel et al., 2021). LTP reduction following pilocarpine is present in the hippocampus, as well as in related structures (Grosser et al., 2020). In this report, as well as in previous publications, we reported that pilocarpine affects the early NMDA-dependent phase of LTP. We showed EPSP elevation in hippocampal slices exposed to thrombin. This effect was suppressed by the PAR1 antagonist SCH79797, and by the NMDAR antagonist ifenprodil. Together, previously published data support early LTP blocking by thrombin through an NMDAR, PAR1 mediated mechanism (Maggio et al., 2008; Lenz et al., 2019). It is interesting to speculate how thrombin can block LTP in a pilocarpine model of SE. In a recent study (Lenz et al., 2019), we have published that thrombin concentrations increase in the hippocampus after pilocarpine SE induction possibly through increased permeability of the BBB. Perhaps BBB permeability increases following seizures, consequently allowing for the entry of thrombin into the hippocampus, activating PAR1 and thus blocking LTP. Further experiments are needed to study this hypothesis. Our results of improved seizure activity upon thrombin pathway inhibition are in line with a previous publication (Lenz et al., 2019), strengthening that inhibition of thrombin has beneficial effects on seizures.

The lower LTP caused by pilocarpine may underlie the behavioral deficits that occur both in humans and in animal models of SE (Beghi et al., 2006; Zhou et al., 2007; Wu et al., 2016). Impairment in learning and memory may indeed persist after experiencing SE (Mikati et al., 2001). Whether thrombin may have a role in this specific aspect is currently not known. One possibility may be that by impairing LTP, thrombin may cause a cascade of events through PAR1 that may lead to enduring behavioral effects. Alternatively, following SE, a permanent leak in BBB may occur therefore resulting in a continuous spillover of thrombin into the hippocampus resulting in a persisting impairment of memory. In this context, it could be interesting to explore whether a modification of PAR1 function through a biased agonism mechanism may prevent the occurrence of a lower LTP induced by thrombin. In a previous set of experiments (Maggio et al., 2013, 2014), we have shown that activation of PAR1 through an activated protein C mechanism may lead to the activation of voltage-gated calcium channels, which counteract the effects of thrombin on LTP in the hippocampus. Thus, may activated protein C injected prior to pilocarpine prevent the decay of the LTP caused by thrombin? Further experiments are needed to test this hypothesis.

Finally, we have shown that upon SE, hippocampal PAR1, but not prothrombin mRNA levels increase. This increase can be modulated by the injection of a PAR1 antagonist. This finding supports the modulation of certain coagulation cascade proteins and calls for specific pharmacological intervention.

In conclusion, in this work, we show that SE impairs LTP in the hippocampus through a PAR1-mediated mechanism. The functional damage upon SE may correlate with increased serum concentration levels of neurofilaments but not with TNF-α levels. Additional experiments are needed to establish the link between neurofilaments concentration and the functional and morphological changes following SE.

## Data Availability Statement

The original contributions presented in the study are included in the article/Supplementary Material, further inquiries can be directed to the corresponding author.

## Ethics Statement

The animal study was reviewed and approved by Institutional Animal Care and Use Committee at the Chaim Sheba Medical Center (#1089-17, #736-12).

## Author Contributions

ES-S and NM: investigation, formal analysis, conceptualization, supervision, data curation, writing—original draft, writing—review and editing. SB: investigation, formal analysis, data curation, writing—original draft, writing—review and editing. TD and UF: investigation. SG: formal analysis, writing—original draft, visualization, writing—review and editing. AD: conceptualization. All authors contributed to the article and approved the submitted version.

## Conflict of Interest Statement

The authors declare that the research was conducted in the absence of any commercial or financial relationships that could be construed as a potential conflict of interest.

## Publisher’s Note

All claims expressed in this article are solely those of the authors and do not necessarily represent those of their affiliated organizations, or those of the publisher, the editors and the reviewers. Any product that may be evaluated in this article, or claim that may be made by its manufacturer, is not guaranteed or endorsed by the publisher.

## Footnotes

^1^ www.graphpad.com
